# Correction: Risk assessment of *Escherichia coli* O157:H7 along the farm-to-fork fresh-cut romaine lettuce supply chain

**DOI:** 10.1038/s41598-025-14501-2

**Published:** 2025-08-06

**Authors:** Ece Bulut, Sarah I. Murphy, Laura K. Strawn, Michelle D. Danyluk, Martin Wiedmann, Renata Ivanek

**Affiliations:** 1 Department of Population Medicine and Diagnostic Sciences, Cornell University, 602 Tower Rd, Ithaca, NY 14853 USA; 2 Department of Food Science and Technology, Virginia Tech, Blacksburg, VA 24061 USA; 3 Food Science and Human Nutrition Department, University of Florida, Lake Alfred, FL 33850 USA; 4 Department of Food Science, Cornell University, Ithaca, NY 14853 USA

Correction to: *Scientific Reports* 10.1038/s41598-025-01585-z, published online 20 May 2025

In the original version of this Article, Figure 2 was incorrectly captured. As a result, panels b and c were missing.

The original Article has been corrected.

